# Vision care in concussion and traumatic brain injury: unmet needs

**DOI:** 10.2217/cnc-2020-0009

**Published:** 2020-07-06

**Authors:** Kenneth J Ciuffreda, Barry Tannen, Penelope S Suter

**Affiliations:** 1 Distinguished Teaching Professor, SUNY/College of Optometry, NY 10036, USA; 2 Emeritus Clinical Professor, SUNY/College of Optometry, NY 10036, USA; 3 Private Practice, Bakersfield, CA 93309, USA

**Keywords:** brain injury, concussion, neuro-optometry, vision care

The world of concussion and traumatic brain injury (TBI) entered a new dimension with the advent of the Iraq and Afghanistan wars, which have persisted over the past 2 decades [1]. This was later further echoed and amplified by the sports concussion ‘epidemic’ [1]. The effect of both occurrences drew much needed attention to the medical condition of concussion/TBI both diagnostically and therapeutically [1], with one emphasis being on the visual sequelae persisting in at least 20% of the patients [2]. Despite this renewed and on-going attention, at least two areas currently have unmet needs: acute concussion detection and clinical intervention for visual sequellae of severe TBI.

The first area of unmet need – namely improved detection and diagnosis of acute concussion – is critical to prevent additional closely spaced, additive concussions [3], especially in our youths. Over the past decade or so, many subjective and objective diagnostic tests have been proposed [4,5]. A subjective test has been the King-Devick oculomotor-based test [6]. The King-Devick test has proven to be a sensitive, noninvasive test, with well-established normative data, which can be administered in the sports field and perhaps in the military theater. However, it is somewhat cumbersome to use, requires several minutes to explain, administer and score, relies heavily on having prior baseline testing done for pre/postcomparison and is best performed by either an experienced professional or a semi-experienced individual in a quiet setting. An objective test is dynamic pupillometry [7–10]. This approach is very rapid (taking about 5 s), noninvasive and easily administered in the sports field or military theater by relatively inexperienced personnel. Additionally, the quantitative results are immediate and have well-established normative data. Unfortunately, despite being a good potential vision biomarker for acute concussion [11] as well as other phases of TBI (e.g., subacute and chronic) [7–10], dynamic pupillometry has not been embraced by many. We suggest that dynamic pupillometry be the first line of diagnostic defense and, if the result is borderline, then the King-Devick test should also be used to improve diagnostic capability (i.e., specificity and sensitivity). Alternatively, both can be used if circumstances permit, which is optimal.

The second area of unmet need is the wider, more aggressive use of simple, therapeutic visual interventions in the severe TBI population [12–14]. Vision care in this population remains a grossly underserved, orphan area. There are many reasons for this, with a likely primary one being the relative difficulty in interacting with this population in the presence of considerable physical, cognitive, attentional and/or language processing deficits, in addition to their constellation of visual dysfunctions. Although this group only accounts for approximately 20% of the overall TBI population [1], it is this subgroup for which the visual system may be a primary means to explore and engage with the world. For example, in the case of a bedridden individual, the eyes may serve as a proxy for their hands and feet. Fortunately, there are several viable and relatively inexpensive options for enhancing quality of life with visual interventions in this population [1,13,14]: objective assessment of the distance and near refractive state using a hand-held autorefractor to assure clarity of vision at all distances with the new spectacle correction; related to #1, the incorporation of a therapeutic tint (e.g., 20% Omega-Brain Power Inc. [BPI]) into the new spectacle correction to relieve the common symptom of photosensitivity; the use of full/partial or opaque/translucent occluders to block the diplopic image in the case of the commonly found TBI-acquired strabismus; related to #3, the incorporation of vergence prisms (e.g., two prism diopters base-in each eye) into the spectacle correction to compensate for the commonly found deficient binocularity and thus reduce the frequency of any transient diplopia; use of a ptosis crutch or other such means to elevate a depressed, paralyzed eyelid [15]; addition of basic, oculomotor-based vision therapy to improve eye tracking ability at all distances and directions, including an eye focusing component in the younger person; and the use of yoked prisms to assist in balance, stance, posture and ambulation by improving the visuo-spatial, egocentric sense [1,13,14]. These visual interventions can be prescribed by a neuro-optometrist or neuro-ophthalmologist, frequently with assistance of the vision therapist or occupational therapist, or in some cases (e.g., in a Veteran’s Administration Hospital) with a specially trained, low vision technician.

The aforementioned areas are two important fields in which we, as vision care professionals, can better serve the concussion and TBI populations. As our knowledge and technology improve, additional diagnostic and therapeutic paths will become available to assist with these and other future unmet needs in the patient with concussion/TBI.
